# Body Acoustics for the Non-Invasive Diagnosis of Medical Conditions

**DOI:** 10.3390/bioengineering9040149

**Published:** 2022-04-01

**Authors:** Jadyn Cook, Muneebah Umar, Fardin Khalili, Amirtahà Taebi

**Affiliations:** 1 Department of Agricultural and Biological Engineering, Mississippi State University, 130 Creelman Street, Starkville, MS 39762, USA; jac1580@msstate.edu; 2 Department of Biological Sciences, Mississippi State University, 295 Lee Blvd, Starkville, MS 39762, USA; mu115@msstate.edu; 3 Department of Mechanical Engineering, Embry-Riddle Aeronautical University, 1 Aerospace Blvd, Daytona Beach, FL 32114, USA; khalilf1@erau.edu

**Keywords:** body acoustics, disease detection, medical conditions, noninvasive diagnosis, sound, vibration

## Abstract

In the past few decades, many non-invasive monitoring methods have been developed based on body acoustics to investigate a wide range of medical conditions, including cardiovascular diseases, respiratory problems, nervous system disorders, and gastrointestinal tract diseases. Recent advances in sensing technologies and computational resources have given a further boost to the interest in the development of acoustic-based diagnostic solutions. In these methods, the acoustic signals are usually recorded by acoustic sensors, such as microphones and accelerometers, and are analyzed using various signal processing, machine learning, and computational methods. This paper reviews the advances in these areas to shed light on the state-of-the-art, evaluate the major challenges, and discuss future directions. This review suggests that rigorous data analysis and physiological understandings can eventually convert these acoustic-based research investigations into novel health monitoring and point-of-care solutions.

## 1. Introduction

**Figure 1:**
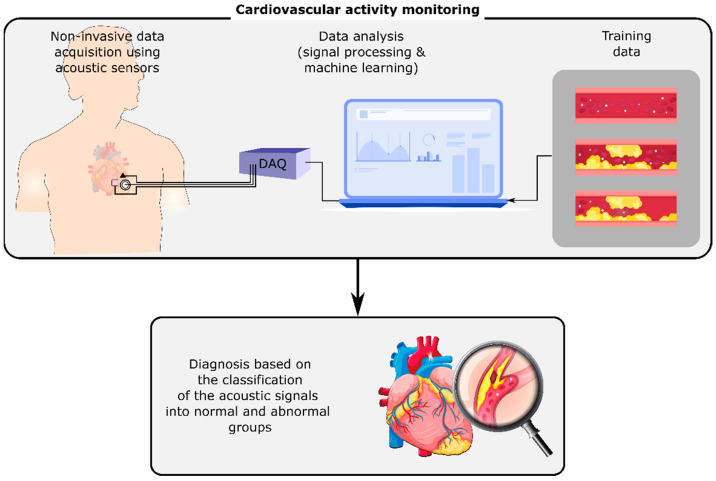
Cardiovascular activity monitoring using the non-invasive measurement of acoustic signals on the chest’s surface. The analysis of these signals using digital signal processing and machine learning methods can identify cardiovascular conditions such as stenosis and aneurysm.

Human body organs and mechanisms generate acoustic signals, including sounds and vibrations, that propagate through the surrounding tissues and reach the body surface. On the body surface, these signals can be measured and monitored noninvasively by acoustic sensors such as microphones, accelerometers, and gyroscopes. They can contain information about the normal or abnormal activity of their sources, i.e., the organs or mechanisms that generated them. For instance, cardiovascular or respiratory-induced sounds can be monitored using a stethoscope on the chest’s surface [1]. Any cardiovascular or respiratory abnormalities may alter these sounds on the chest, e.g., by changing the sound signals at their origin or the material properties of the tissues that the signals pass through. The variations of these signals can be investigated in order to detect and identify the abnormality (Figure 1). Advancements in wearables and sensing technology, coupled with the developments in computing, signal processing, and machine learning methods have enabled the long-term, real-time measurement and analysis of these signals even outside of healthcare facilities, which may result in the earlier diagnosis of diseases and the timely referral of the patients to their caregivers.

This paper reviews the diagnosis and monitoring methods that are being developed based on body acoustics for different types of medical conditions. An in-depth and comprehensive search of the Web of Knowledge, Embase, and MEDLINE databases was carried out with the combination of the following terms: “sound”, “vibration”, “acoustics”, “cardiovascular system”, “circulatory system”, “gastrointestinal system”, “digestive system”, “nervous system”, “respiratory system”, and “noninvasive diagnosis”, from 1980 to 2021. As a result, the following general categories were selected to be included in this review: cardiovascular diseases, respiratory illnesses, gastrointestinal diseases, and nervous system disorders. Such a review of the state-of-the-art provides a larger picture of the current challenges and potential future directions of the acoustic-based point of care diagnosis methods. Our review suggests that biomedical acoustic signals have a high potential to detect different types of medical conditions, especially by the remote monitoring of patients and high-risk individuals. However, there are still open questions and challenges that hinder the translation of these methods from a research interest to a powerful and reliable clinical monitoring tool.

## 2. Cardiovascular Diseases

One in every four Americans dies due to cardiovascular diseases (CVDs) every year, which makes CVDs the leading cause of death in the United States [2]. The earlier detection of the potential signs of the disease would make early treatments possible, and may prevent subsequent cardiovascular complications [3]. Despite many efforts, the development of novel robust diagnosis methods is still an urgent need for the earlier detection of many types of CVDs. This may become possible through the more-frequent evaluation of the patient’s status, for example, by low-cost user-friendly remote monitoring methods that can be used outside of the healthcare facilities. The current commercial remote monitoring devices are mainly based on electrocardiography (ECG). However, some CVDs such as structural abnormalities in heart valves may not be clearly seen in the ECG signal because they do not change the electrical depolarization of the myocardium [4]. In the research setting, other methods such as cardiovascular-induced sounds and vibrations on the chest’s surface have been also widely investigated [5,6,7]. These signals provide information about the mechanical aspects of cardiovascular activity, and thus may contain diagnostic information that is complementary to the existing methods such as ECG.

After these signals are measured using the sensing systems, they are usually analyzed using feature extraction and machine learning methods to classify them into healthy and diseased groups. For example, the temporal and spectral contents of cardiovascular sounds have been explored by methods such as wavelet transform, power spectral density, and mel-frequency cepstrum [8,9,10,11]. The extraction of the time and frequency features of the heart vibrations has been performed through short-time Fourier transformation, wavelet transformation, Wigner-Ville distribution, and polynomial chirplet transformation [12,13,14,15]. The acoustic signals can be also decomposed into their components using methods such as ensemble empirical mode decomposition and variational mode decomposition [16,17]. Additional features can be extracted from the resulting modes, or these modes can serve as signal features themselves. These decomposition methods can also assist with signal filtration by excluding the modes which are believed to be related to noise. Furthermore, some studies have suggested that the inclusion of respiration information would organize the signal features into separate groups that could lead to the more accurate classification of heart vibrations into normal and abnormal groups [18,19,20,21,22,23]. Throughout the different phases of the respiration cycle, there is variation in the temporal and spectral characteristics of heart-induced vibrations. For example, after segmenting vibration signals into smaller segments where each one represents one cardiac cycle, the segments that occur during the high-lung-volume phase are different from those during the low-lung-volume phase in both the time and frequency domains. This suggests that breaking the heart vibration signals into two different groups based on the respiration cycle information can result in a better signal characterization [19]. Other popular signal processing and analysis methods of the cardiovascular-induced sound and vibration signals have been reviewed in previous work [5,6,24,25,26].

In recent studies, cardiovascular vibrations played a vital role in the development of methods for the monitoring of cardiovascular activity and the early detection of irregularities. The axial and torsional components of these vibrations may be recorded noninvasively on the chest surface using sensing systems that include accelerometers and gyroscopes. These axial and torsional vibrations are called seismocardiograms (SCG) [27,28] and gyrocardiograms (GCG) [29,30], respectively. These precordial vibrations are generated by cardiac movements such as heart contraction, the opening and closure of the heart valves, and blood flow momentum and turbulence [5,6]. SCG and GCG signals can be measured from different locations on the chest. Some of the common measurement locations include the sternum, the right upper sternal border, and over the heart apex [5]. Other popular vibration-based cardiovascular activity monitoring methods include ballistocardiography [31,32], vibrocardiography [33], kinocardiography [34], and mechanocardiography [35]. These noninvasive cardiac-health monitoring methods have a duality in their applications; specifically, they have use in a clinical setting as well as in the home.

Cardiac vibration signals can provide information about the heart rate and cardiac cycle duration. Each cardiac cycle is composed of two main segments: the systolic and diastolic phases. In one cycle, the systolic portion is usually shorter than the diastolic portion [36]. In addition, the duration of the cardiac cycle depends on the respiration phase in which the cardiac cycle occurred [22]. The variations in the heart rate can be measured from the cardiac cycle duration, which can be determined by heart vibration signals [22,37,38]. These variations are known as predictive factors of different CVDs, such as sudden cardiac death [39]. Furthermore, the temporal features of the heart activities such as cardiac time intervals and fiducial points (e.g., the opening and closure time of the cardiac valves) can be also determined and analyzed using heart vibration signals [40,41,42]. These measurements assist in providing insight into the functions of the heart. For example, a recent study estimated the cardiac time intervals from impedance cardiography, PCG, and SCG [43]. The estimations of each method were compared with the gold standard values that were measured by multimodal echocardiography. The results suggested that SCG had a significantly higher accuracy than the other methods for the estimation of the pre-ejection period and the total systolic time.

Cardiovascular vibrations assisted in the diagnosis and monitoring of different clinical conditions. Earlier studies have been conducted to measure the heart motion of anesthetized pigs during surgery using a three-axis accelerometer [44]. The accelerometer output was filtered at 1 Hz in order to retain the movements due to the heart beating and remove the low-frequency respiration vibrations. The results suggested that accelerometers could locate irregular patterns that may indicate heart circulation failure—a sign of numerous CVDs. In another early study on human subjects, the functioning of the left ventricle has been observed through signals derived from SCG in order to detect myocardial ischemia [45]. Alterations in cardiac muscle contractions were noted and analyzed. The findings were conclusive enough to be used to diagnose patients with ischemia. More recent studies have used cardiac vibrations to diagnose medical conditions such as heart failure [46,47], coronary artery disease (CAD) [48], valvular disease [49], ischemia [50], atrial fibrillation [50,51,52], atrial flutter [53], aortic stenosis [54,55], and myocardial infarction [56]. Further uses of left ventricular monitoring using SCG have been also proposed in areas such as cancer patients’ cardiotoxicity assessments [57].

**Figure 2:**
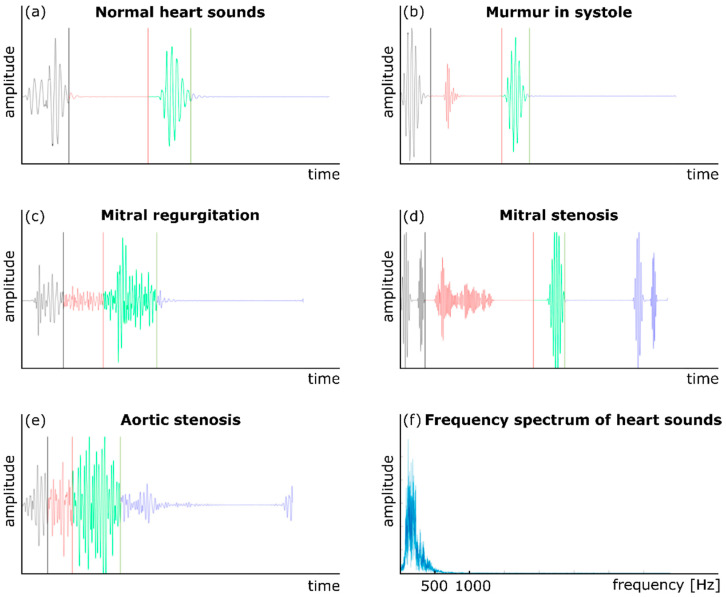
Normal and abnormal heart sounds: (**a**) a normal heart sound, (**b**) murmur in the systole, (**c**) mitral regurgitation, (**d**) mitral stenosis, (**e**) aortic stenosis, and (**f**) the frequency content of a PCG signal. Adapted from ref. [10].

Auscultation has also been used to monitor cardiac health and activity for many years. Phonocardiography (PCG) is another common technique to record cardiovascular acoustics using a traditional or digital stethoscope [10,26]. In addition to normal heart sounds (S1 and S2), a PCG signal may contain components that indicate pathological conditions. For example, the presence of a third heart sound, S3, which occurs right after S2, can be a sign of congestive cardiac failure [58]. Figure 2 shows five sample normal and abnormal PCG signals. The presence of a cardiovascular abnormality or malfunction such as an aneurysm or stenosis may create turbulence in the downstream blood flow in a vessel [59,60]. The turbulent flow generates sounds in specific frequency bands (depending on the morphology and severity of the abnormality) that transmit to the vessel wall, then to the surrounding tissues, and eventually to the body surface. These variations may then be analyzed using signal processing and machine learning methods to detect the abnormality. For example, CAD is caused by large amounts of plaque deposit buildup in the coronary arteries, which limits blood flow. Currently, the most accurate way to test for CAD is coronary angiography, which is both invasive and expensive. Non-invasive ways to detect CAD include low-cost methods that are based on the non-invasive measurement of heart sounds using digital stethoscopes [61]. In order to diagnose CAD, one study obtained the diastolic sound segments by stethoscope in CAD patients and analyzed them using a multivariate classifier [61,62]. Subtle differences were noted between the diastolic sound from healthy individuals and patients with CAD, which suggested that a digital stethoscope has potential for noninvasive CAD diagnosis.

Computational fluid dynamics and post-processing numerical techniques are other effective, noninvasive approaches to analyze the sounds emitted from the human body [63,64]. Acoustic pressure fluctuations have been recognized as the primary flow-induced source of the sound through arteries and lung airways. These pressure fluctuations can be further excited by blood flow turbulence due to medical conditions such as stenosis and aneurysm. The pressure fluctuations are correlated with highly turbulent shear stresses and vibration on the internal arterial wall leading to the structural response of the surrounding tissue. Computational fluid dynamics simulations can investigate the sounds coming from highly turbulent fluctuating flow through the arteries, and explain the mechanisms of the clinical observations. This provides information that can help predict a variety of cardiovascular diseases [59,60]. In the past studies, vessel wall shear stress and pressure were used as indicators of an unhealthy artery with a high potential risk of complications leading to abnormal sound signals propagated through the tissue [65,66,67,68,69,70]. In these studies, adverse hemodynamic conditions at critical times during a heart cycle were investigated to find the correlation of transient pressure fluctuations due to highly turbulent kinetic energy with structural vibrations and stresses associated with elevated risk of hemolysis, platelet activation, and potential development of thrombosis. It was concluded that the highest turbulent stresses through an unhealthy artery can be 4–14% higher than the stresses through a healthy artery at peak systole. Moreover, the results from the pressure fluctuations and stresses on the internal arterial wall were utilized to analyze structural vibrations in the frequency domain, and to understand the mechanism of sound propagation to the epidermal surface. The acquisition of the sound signals on the chest surface propagated from unhealthy arteries can be used as a noninvasive diagnostic tool to identify the disease. Therefore, the post-processing of the sound features and their spatial distribution can contain valuable information about the geometric details of the disease, which can be essential for the diagnosis process. These studies post-processed the time-series of the pressure or wall shear stress that were recorded on the vessel wall in order to analyze the frequency contents of the flow fluctuations.

The information supported by decomposing the time-varying flow field into spatial and temporal parts through proper orthogonal decomposition (POD) can also help provide insights into the acoustic sources in the flow [59,60]. The decomposed time-varying flow can be reconstructed to describe the modes of coherent flow structures as sound sources and the time evolution of these modes. Additionally, POD analysis followed by a frequency-based temporal filtering method can visualize the coherent structures at specific frequencies to provide useful information on the localization of sound sources with maximum sound pressure levels through unhealthy systems. Additional information on the process of the POD method can be found in [59,60]. It should be noted that the importance of the accurate post-processing of CFD results with state-of-the-art methods such as the POD has been highlighted in previous studies. For example, it is suggested that POD can reconstruct features of complex recirculating flows through small vessels [71], while the spatial resolution of 4D-flow MRI is not sufficient to resolve these flow features accurately [72]. POD has also been used to study the flow through mechanical aortic valves [73], cerebral aneurysm [74,75], and coronary arteries [76].

## 3. Respiratory Illnesses

For centuries, simple and low-cost acoustic procedures such as auscultation, percussion, and tactile fremitus have allowed physicians to evaluate and address respiratory system diseases [77,78]. With advances in sensing technologies and the aid of advanced signal processing techniques, the more reliable feature extraction and classification of the respiratory sounds and vibrations have been developed, leading to a better understanding of both normal and abnormal lung acoustics. In order to classify signals that differentiate disease from health in a person, efficient feature extraction is needed. Different methods have been employed to properly extract features from lung sounds and vibrations in the time, frequency, and time–frequency domains. Examples of these would be Fourier transformation, mel-frequency cepstrum, wavelet transformation, and Hilbert–Huang transformation [77].

Various locations on the body produce respiratory sounds which need different measurement techniques and devices. For example, airway impedance is measured from the lips [79]. In other applications, respiratory acoustics would be recorded from the neck or chest surface. When the signals are measured on the neck (e.g., at the suprasternal notch over the extrathoracic trachea), they present larger amplitudes than recordings from the chest [80,81,82]. Measurements from the neck cover frequencies from 80 Hz to 1500 Hz, which includes the frequency range of many diseases (Table 1). In general, the frequency range of interest for the analysis of lung sounds is 100–2000 Hz. Because the chest behaves as a low-pass filter, it hinders higher-frequency components [77]. The frequency components below 100 Hz are also usually eliminated in order to remove the acoustic signals from other sources such as the heart, muscles, and electrical inference.

Acoustic signals have been used to study the respiratory system in health and disease. For example, vibration response imaging (VRI) revealed variations in respiratory-induced vibrations between smoker and non-smoker individuals in healthy asymptomatic subjects [87]. VRI is a 2D grayscale imaging technique that measures respiratory acoustic variations using an array of piezo-acoustic sensors on the body’s surface [88]. Furthermore, a wide range of acoustic-based methods have been investigated and developed to detect respiratory conditions including asthma [1,89,90,91,92,93,94], obstructive sleep apnea [95,96,97], chronic obstructive pulmonary disorder [1,93,98,99,100,101], tracheal stenosis [102], pneumothorax [86,103,104,105], pneumonia [1,106,107,108], pleural effusion [109], cystic fibrosis [110,111], and COVID-19 [112,113]. Some of these methods and medical conditions will be discussed in more detail in the rest of this section.

Spirometry is currently one of the most common procedures which is performed to evaluate pulmonary function [114]. This test utilizes sound waves that come from the patient’s breathing patterns to diagnose respiratory diseases. During this procedure, the patient breathes deeply and slowly into a mouthpiece that is connected to a spirometer. This technique, however, can be limited by the patient’s ability to perform the test accurately, making it increasingly difficult for elderly people and younger children [77].

Due to these limitations, there have been alternative methods to diagnose children with pulmonary medical conditions such as asthma. For example, the forced oscillation technique uses tidal breathing, which requires little effort from children. This technique requires subjects to be in a seated position with straight backs and neutral or extended neck positioning [115]. In addition to the subject’s positioning, the cheeks and floor of the mouth should be supported by another person or physician. The forced oscillation technique is commonly tested using a mouthpiece that features a bacterial filter and a nose clip-on. In order to detect asthma using this technique, the system analyzes the relationship between the airflow and airway pressure from a respiration cycle (respiratory resistance and reactance). Another way to use bioacoustics to diagnose respiratory diseases is the use of tracheal sounds [102], which can be proven in people who suffer from obstructive sleep apnea [116]. Obstructive sleep apnea is a sleep disorder in which breathing is disrupted for longer than ten seconds and at least five times per hour throughout a single sleep period. In this case, the tracheal sounds come from the surface vibrations on the suprasternal notch. Acoustic-based sensors are able to track the pressure changes produced by the mass, elastance, and resistance of the trachea [116]. These sensors are inserted into a protective plastic chamber with a deep cuff. This creates an airtight space between the transducer and the skin. Adhesive tape is also usually used to keep the sensor placed right above the suprasternal notch and ensure a good sensor–skin coupling.

**Figure 3:**
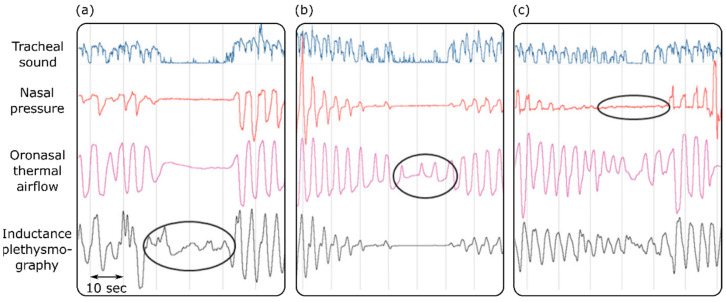
Detecting sleep apnea using tracheal sounds, nasal pressure, oronasal thermal airflow, and inductance plethysmography. Apnea is not detected by inductance plethysmography or the oronasal thermal airflow sensor in (**a**,**b**). Oral breathing is mistaken for apnea by the nasal pressure transducer (**c**). Adapted from ref. [118].

Sleep apnea is defined as a condition where the airflow is reduced by more than 90% for more than 10 s in an adult subject [117]. Different airflow techniques have been used for the diagnosis of obstructive sleep apnea in sleep laboratories and clinics [118,119,120]. These techniques included respiratory inductance plethysmography, oronasal thermal airflow measurements, and nasal pressure measurements. One study assessed the effectiveness of tracheal sound measurements in the detection of apnea [118]. The results suggested that tracheal sound measurements are efficient and reliable enough to accurately pick up and provide a noninvasive way to diagnose obstructive sleep apnea. Furthermore, the tracheal sound signals could detect apnea on occasions when it was missed by the other diagnostic techniques (Figure 3). Because apnea affects the oxygen delivery to different organs, it causes a muscle metabolic reflex, which in turn affects the blood pressure and cardiac contraction [119]. Based on this, it is suggested that maximum expiratory apnea increases the kinetic energy of the heart, which can be measured using cardiac vibration signals such as SCG and BCG [120].

Pneumonia is the result of a bacterial or viral infection that successfully inflames the lungs and converts the air sacs to a solid as they fill with pus. This disease can be very serious, or even fatal [121]. Imaging techniques such as chest X-rays are the most common ways to diagnose pneumonia. However, pneumonia is often unclear in these images, which may result in misdiagnosis [122]. Once the disease is detected, there are various treatments to reduce the effects of pneumonia in patients. The outcome from these treatments needs to be tested in order to find out how efficient they are the successful reduction of pneumonia. X-ray tests are currently the method of choice; however, these tests are expensive and impractical due to the excess amount of radiation applied to the patient. Researchers have begun to explore alternative approaches, one of which is VRI [93]. This technology focuses on lung function by recognizing the relationship between the gas flow in the lungs and the vibration energy. This process requires patients to be seated facing the VRI machine with multiple sensors placed on the back while respiratory cycles are recorded [77]. The placement of sensors varies from study to study [123]. For example, one study used two V-array sensors placed on the back, 1.5 cm above the mesoscapula [124]. They were positioned in parallel from right to left, with 5 cm allocated for a spin on the two sides. There were three to five, 12-s cycles of natural breathing that were recorded for the acquisition of data. After testing 62 subjects, the drastic change (*p* < 0.001) in the VRI images before and after treatment proved that this new VRI technology was successful in the evaluation of curative effects for pneumonia.

Chronic obstructive pulmonary disease (COPD) is a group of illnesses with the potential to block airflow and create problems related to the ability to breathe [125]. It is currently the third leading cause of death in the world [126]. One way to diagnose COPD is using a stethoscope, the accuracy of which relies on there being no human error in the dissection of the auscultations [1]. Newly developed digital stethoscope increase the reliability in diagnosis substantially [77]. By combing the conventional stethoscope with computerized systems, the digital stethoscope could produce an efficient technique for the discovery of common COPD conditions such as emphysema and chronic bronchitis [127,128]. Other studies have been carried out to test the accuracy of digital stethoscopes in the diagnosis of various respiratory illnesses [129]. However, these devices may also capture the surrounding noise signals, which should be carefully removed [130].

In addition to the assessment of respiration acoustics using sensing systems, numerical models have been used to investigate the sound transmission and vibration propagation within the respiratory system [131]. Clinical investigations of flow through the lung airways are challenging because of the complex geometry of lung airways, the transient nature of the airflow during the respiration cycle, and difficulties in locating small obstructions induced by the chronic pulmonary diseases in the medical images [132,133]. Numerical models can provide a comprehensive analysis of flow, which can potentially help explain airflow complications and support medical decision-making. This detailed information may not, however, be obtained with in-vivo measurements. For example, Hu et al. [132] demonstrated an innovative noninvasive diagnostic method using computational modeling and neural network techniques to localize the small obstructions in the peripheral lung based on the flow velocity contour shifts. In another study, the effects of different severity levels of upper airway deformation on the airflow structure were studied numerically in order to show the existence of turbulence flow and an increase in the flow fluctuations [134]. Although these numerical models can provide detailed information on respiratory flow, they are computationally expensive and usually require a few hours or days of processing time.

## 4. Other Diseases

**Figure 4:**
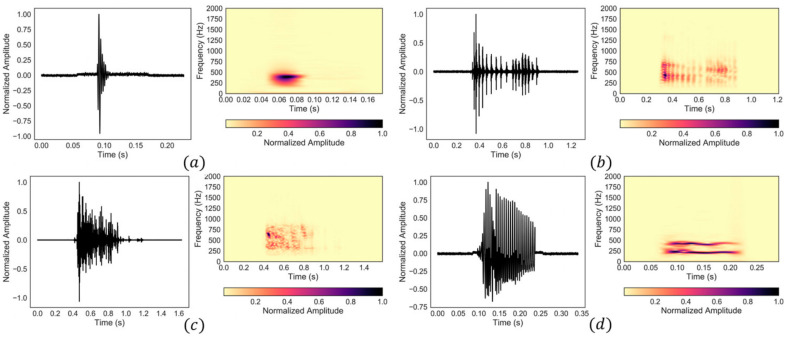
Four common types of bowel sounds: (**a**) single burst, (**b**) multiple bursts, (**c**) continuous random sound, and (**d**) harmonic sound. Reprinted from ref. [135].

Biomedical acoustics have been also used to develop monitoring methods for other medical conditions. For instance, the digestive system generates sounds and vibrations that can be used for diagnosis purposes. Bowel sounds can be categorized into four typical types based on their waveform and frequency content [135]. These include single burst, multiple bursts, continuous random sound, and harmonic sound types (Figure 4). Alternatively, considering time-frequency features, abdominal sounds and vibrations can be classified into two groups of intestinal bursts and regularly sustained sounds (short and long duration sounds, respectively) as well as three sets of interfering noise, including respiration, snoring, and motion-related noise sets [136,137]. Table 2 lists the time and frequency signatures of different types of bowel sounds. Although some studies suggest that bowel sounds do not provide clinically relevant information [138], other studies have used the noninvasive monitoring and analysis of stomach and bowel sounds for the diagnosis of gastrointestinal tract diseases. These medical conditions include abdominal conditions such as appendicitis, intestinal obstruction, irritable bowel syndrome, inflammatory bowel disease, pyloric stenosis, postoperative ileus, and cholecystitis [139,140,141,142,143,144,145,146]. Despite respiratory and cardiac acoustics, the monitoring of gastrointestinal acoustics usually requires continuous data acquisition of several minutes, as gastrointestinal sounds do not occur frequently [147].

Bioacoustics sensors were also used to monitor the conditions of patients with nervous disorders such as Parkinson’s disease [148,149] and multiple sclerosis [150]. For example, accelerometers can evaluate the movement data of patients with multiple sclerosis, a chronic neurological disease. These data can be then used to assess the daily activities of the patients and provide clinical feedback in order to manage the symptoms of multiple sclerosis [150]. Tremor is the involuntary and rhythmic oscillatory movements of a body part, and is a symptom of many disorders, including cerebellar disease, peripheral neuropathy, orthostatic tremor, and Parkinson’s disease [151]. The frequency of the tremor can range between 3 Hz (slow) and 12 Hz (rapid). These frequencies and the amplitude of the tremor can be continuously monitored using wearable accelerometers in order to evaluate the severity of the disease [152]. Other studies have used acoustic signals for the non-invasive diagnosis and monitoring of other medical conditions, including voice disorders (e.g., phonotraumatic vocal hyperfunction) [153,154], dysphagia [155,156,157], fetal health and development [158], and cartilage-based conditions such as osteoarthritis and chondromalacia [159,160].

## 5. Conclusions and Future Directions

Advances in the development of lighter, cheaper, and more sensitive and accurate sensors have resulted in a resurgence in research on body acoustics for the non-invasive diagnosis of different medical conditions. In addition, the recent improvements in computational resources have made the real-time continuous monitoring of the patients a reality inside and outside of clinical settings [31,161,162]. These developments can lead to the formation of large databases of body acoustics. The analysis of this big data using methods such as deep learning may provide a better understanding of the body acoustics, and may establish novel non-invasive diagnosis methods. These signals may also provide complementary information to other monitoring methods. For example, while an ECG test assesses the electrical activity of the heart, acoustic signals such as sounds and vibrations can provide a more in-depth understanding of the mechanical activity of the cardiovascular system. This complementary information can lead to the detection of early signs of CVDs.

Acoustic signals may also assist in the non-invasive localization of the abnormalities inside the body [163]. Simultaneous measurements of acoustic signals from multiple locations on the body surface using sensor arrays can provide information on the 3D location of the acoustic source (e.g., cardiovascular diseases or lung pathologies). For example, the location of heart valves may be estimated by the analysis of the outputs of a microphone array [106,164].

Despite the many studies on the utility of acoustic signals for medical diagnosis, there are still open questions and challenges that hinder the transformation of these research studies into commercial products. One of the challenges in the use of acoustic signals for medical diagnosis is the presence of different noise sets in the signal of interest. The acoustic signals that are recorded on the body’s surface consist of sounds and vibrations from other sources that are not of interest. For example, a fetal PCG is a mixture of acoustic signals from the fetus, different organs of the mother, and other external noises [158]. In addition, any fetal movements can add an artifact to the fetal PCG. On the other hand, the acoustic signals recorded from a pregnant woman are contaminated by signals that originated from the fetus. Robust noise filtration methods should be established in order to isolate the signals of interest from noise.

Another challenge is that the temporal and spectral contents of the acoustic signals depend strongly on the acquisition location. For example, heart-induced vibrations on the chest’s surface may change by 30% with a sensor location change of 1 cm [5]. Other parameters such as the postural position and transmission medium (e.g., the amount of soft tissue between the signal source and the sensor) also affect the contents of the acoustic signals. In addition, these signals usually change from one subject to another. Therefore, the analysis methods should be developed such that they are robust, inclusive, and accurate under different conditions.

In conclusion, the utilization of new generations of sensing technologies in combination with signal processing methods and rigorous experiments to understand the physiological genesis of the acoustic signals may translate them from a research interest to a powerful tool for the diagnosis of medical conditions.

## Figures and Tables

**Table 1:** Frequency signatures of respiratory diseases.

Disease	Frequency Range [Hz]
Pneumonia	300–600 [1,83]
Asthma	165 [84] 239 [4] 329 [85]
Chronic obstructive pulmonary disorder	233–311 [85]
Pneumothorax	400–600 [86]

**Table 2:** Time and frequency signatures of different types of bowel sounds. Data from ref. [135].

Type	Duration [ms]	Spectral Centroid [Hz]
Single burst	18–58	347–681
Multiple bursts	100–1030	345–753
Continuous random sound	119–1637	316–609
Harmonic sound	73–763	269–630

## Data Availability

Not applicable.
